# Psychometric evaluation of Azeri version of the head and neck cancer specific quality of life questionnaire (EORTC QLQ-H&N43)

**DOI:** 10.1186/s12955-020-01500-2

**Published:** 2020-07-23

**Authors:** Mahammad M. Davudov, Chingiz Rahimov, Iraj Harirchi, Zoheir Mirzajani, Namig Amiraliyev, Kanan Amiraliyev, Narmin Rustamova, Jayran Zebardast, Ali Montazeri

**Affiliations:** 1 Cancer Institute, Tehran University of Medical Sciences, Tehran, Iran; 2 Department of Oral and Maxillofacial Surgery, Azerbaijan Medical University, Baku, Azerbaijan; 3 Department of Oncology, Azerbaijan Medical University, Baku, Azerbaijan; 4 Population Health Research Group, Health Metrics Research Center, Iranian Institute for Health Sciences Research, ACECR, Tehran, Iran; 5 Faculty of Humanity Sciences, University of Science and Culture, ACECR, Tehran, Iran

**Keywords:** Psychometric analysis, EORTC QLQ-H&N43, Azerbaijan, Oral cancer

## Abstract

**Background:**

Oral cancer surgery can have a deep effect on the quality of life in the patient both in terms of functional and psychological aspects. This study aimed to translate and validate the European Organization for Research and Treatment of Cancer head and neck cancer specific quality of life questionnaire (EORTC QLQ-H&N43) in Azerbaijan.

**Methods:**

Forward-backward translation was applied in order to translate the EORTC QLQ-H&N43 from English into Azeri. Then, a sample of patients with oral cancer attending a teaching hospital affiliated to Azerbaijan Medical University completed the EORTC QLQ-C30 (the core cancer specific questionnaire), and the EORTC QLQ-H&N43. To evaluate psychometric properties of the QLQ-H&N43, known groups validity, convergent and divergent validity was performed. Internal consistency reliability was examined by estimating the Crornbach’s alpha coefficient.

**Results:**

Ninety-six patients with confirmed diagnosis of oral cancer were entered into the study. The mean age of patients was 59.6 (SD = 10.7) years and 36 patients (37.5%) diagnosed as having stage IV and 10 patients (10.5%) were metastatic. The results obtained from comparing quality of life scores among these patients showed that the questionnaire was able to differentiate among patients who differed in stage and metastasis lending support to its validity. In addition convergent and divergent validity showed satisfactory results. The internal consistency of the multi-item scales as assessed by the Cronbach’s alpha coefficient showed acceptable results (alpha ranging from 0.66 to 0.78).

**Conclusion:**

The findings suggest that in general the Azeri version of EORTC QLQ-H&N43 has satisfactory internal consistency reliability and validity, but additional psychometric evaluation is needed to draw firm conclusions.

## Background

Oral cancer and its treatment have substantial effect on patients’ quality of life [1]. More importantly there are several factors that deepen this effect. For instance patients with oral cancer who receive surgery finds problems with appearance, speech, ability to breathe, eat and swallow [2]. All these could have both short- and long-term effect on patients’ physical, mental and social well-being [3]. Thus in clinical settings it is important to assess health-related quality of life in this population in order to evaluate treatment outcomes. As such questionnaires that have been developed by the European Organization for Research and Treatment of Cancer (EORTC) are very well known and are frequently used in outcome studies in oncology. For instance the EORTC QLQ-C30 was developed for measuring quality of life in all cancer patients (core cancer questionnaire) [4] and consequently for many anatomical sites specific instruments proposed to be used in conjunction with core questionnaire and currently they are in use worldwide. One of these instruments is the specific questionnaire that was developed for measuring quality of life in patients with head and neck cancer. The questionnaire first introduced with 35 items in 1999 and was named the EORTC QLQ-H&N35 [5, 6]. Since then the QLQ-H&N35 was translated and validated in different countries and a number of various languages including US English [7], Italian [8] Taiwan Chinese [9], Japanese [10], German [11], Cantonese (Hong Kong) [12], Greek [13], Chinese [14], Mexican Spanish [15], and Arabic [16].

However later on after comprehensive review by the EORTC Quality of Life Group and the EORTC Head and Neck Cancer Group it was updated and it was tested in Danish, Dutch, French, German, Greek, Hebrew, Italian, Japanese, Mandarin, Norwegian, Polish, Portuguese, Spanish, and Swedish following forward-backward translation of original English version of the questionnaire. This update version of the questionnaire contains 43 items and examines several important symptoms in head and neck cancer patients [17].

Since the Azeri version of the questionnaire was not available we aimed to translate and validate the QLQ-H&N43 questionnaire in Azerbaijan.

## Methods

### QLQ-H&N43

The EORTC QLQ-H&N43 is a supplementary module for the EORTC QLQ-C30 that measures quality of life in patients with had and neck cancers. It contains 43 items tapping into 6 multi-item and 13 single-item symptom subscales namely pain, swallowing, senses problems, speech problems, trouble with social eating, less sexuality, teeth, dry mouth/sticky saliva, body image, shoulder pain, skin problems, anxiety, trouble with social contact, opening mouth, coughing, lymphedema, problems wound healing, weight loss, neurological problems [17]. Each item is rated on a 4-point Likert scale and scores for each subscale ranges from 0 to 100 where higher scores indicate greater symptoms.

### Translation

After asking permission from the EORTC Quality of Life Study Group, forward-backward procedure was applied to translate the questionnaires from English into Azeri. Two independent translators translated the original questionnaire into Azeri. Although there were some differences between two translations, the research team dealt with these differences and provided a single forward version of the questionnaire. For instance there were differences in translating skin ‘rash’ or ‘skin changed color’. Consequently two other bilingual translators back translated the questionnaires from Azeri into English. Accordingly a single back translated version of the questionnaire made available and it was checked with the original questionnaire for any errors or deviations. To check cultural relevance of the translation, and ease of comprehension five patients (not included in the current study) the instrument was pre-tested. However, since it was verified, the provisional version of the Azeri questionnaire was subjected to psychometric properties.

### Psychometric evaluation

A cross sectional study was conducted on a sample of patients with confirmed oral cancer attending a teaching hospital affiliated to Medical University of Azerbaijan in Baku from year 2011 to 2017. They all completed the study questionnaires including the Azeri version of the EORTC QLQ-C30 and the EORTC QLQ-H&N43.

### Statistical analysis

Validity was examined using known groups comparison. We hypothesized that the questionnaire should be able to differentiate between patients who are differing in stage and metastasis. In fact we hypothesized that patients with lower stage and no metastasis should score better than patients with higher stage and having metastasis. For comparison the Mann-Whitney and Krurskal-Wallis tests were used. In addition convergent and divergent validity (discriminant validity) was examined by estimating correlation coefficient between the EORTC QLQ-C30 and the EORTC QLQ-H&N43 scores. Due to skewed distribution of the data we used the Spearman’s rho coefficient. Correlation coefficients ranging 0.1–0.3 were considered low, 0.31–0.5 as moderate, and those exceeding 0.5 as high. Internal consistency reliability for multi-items subscales (having at least 3 items) was assessed using the Cronbach’s alpha coefficient. Alpha coefficient equal or greater than 0.7 was thought satisfactory.

## Results

A total of 96 patients (67 men and 29 women) who underwent flap reconstruction for oral cancer in Azerbaijan were studied. The mean age of patients was 59.6 (SD = 10.7) years ranging from 30 to 82. In 47 cases, age was lower than 60 years. The characteristics of patients are given in Table 1. In addition the descriptive quality of life scores for all patients including the floor and ceiling effects are presented in Table 2.

**Table 1:** Demographic and clinical characteristics of the study sample (*n* = 96)

	Frequency	Percent
**Gender**		
Male	67	69.8
Female	29	30.2
**Age**		
< 60	49	51
≥ 60	47	49
**Tumor grade**		
1	16	16.7
2	27	28.1
3	30	31.2
4	23	24
**Nodes**		
0	65	68
1	13	13.5
2	11	11.5
3	7	7.3
**Metastasis**		
No	86	89.6
Yes	10	10.4
**Radiotherapy**		
No	56	58.3
Yes	40	41.7
**Stage**		
I & II	34	35.4
III	26	27.1
IV	36	37.5
**Chemotherapy**		
No	75	78.1
Yes	21	21.9

**Table 2:** The EORTC QLQ-C30 and the QOL-H&N43 scores for the patients (*n* = 96)

	Mean	Standard deviation	Floor/ceiling effects (%)
**QLQ-C30**			
*Functioning*^*a*^			
Physical functioning	90.5	13.7	0.0/12.7
Role functioning	83.8	16.8	0.0/15.4
Emotional functioning	72.1	17.0	0.0/5.4
Cognitive functioning	86.5	20.2	0.0/9.7
Social functioning	80.7	20.5	0.0/12.8
Global quality of life	61.7	14.4	1.1/12.0
*Symptoms*^*b*^			
Fatigue	27.1	22.6	11.0/2.2
Nausea and vomiting	8.51	17.2	3.3/13.0
Pain	33.3	19.2	7.6//9.8
Dyspnoea	16.6	22.3	14.4/10.8
Insomnia	12.3	23.5	8.6/1.1
Appetite loss	18.1	24.9	8.0/15.2
Constipation	34.0	30.4	3.7/6.5
Diarrhoea	23.9	28.0	8.9/4.3
Financial difficulties	37.6	26.7	1.1/25.0
**QLQ-H&N43**^**b**^			
HN Pain	29.5	22.4	9.9/1.1
HN Swallowing	29.6	16.4	4.3/5.4
HN Senses problems	15.0	18.3	6.5/2.2
HN Speech problems	26.3	16.7	5.4/1.1
HN Trouble with social eating	28.0	18.2	10.9/0.0
HN Less sexuality	21.4	27.3	4.9/2.2
HN Problems with teeth	44.5	25.1	13.0/5.4
HN Dry mouth/Sticky saliva	11.0	19.8	8.5/1.1
HN Body image	24.8	21.1	13.9/0.0
HN Shoulder pain	5.49	15.5	7.3/0.0
HN Skin problems	7.65	17.0	12.0/1.1
HN Anxiety	48.3	30.1	17.8/5.7
HN Trouble with social contact	12.4	24.6	6.6/2.2
HN Opening mouth	31.5	32.1	13.4/6.5
HN Coughing	11.2	23.3	14.1/2.8
HN Lymphedema	9.52	18.7	15.8/0.0
HN Weight loss	27.0	31.5	14.4/1.1
HN Problems wounds healing	12.9	21.6	12.1/4.4
HN Neurological problems	11.1	21.1	13.8/2.2

^a^Higher scores indicate better conditions

^b^Higher scores indicate greater symptoms

### Validity of the QOL-H&N43

#### Known groups validity

As shown in Tables 3 and 4 although not significant, the QLQ-H&N43 differentiated patients who differed in stage and metastasis. As expected patients with the advanced disease scored higher almost on all symptoms and thus lending support to the validity of the questionnaire.

**Table 3:** The QLQ-H&N43 scores in patients with and without metastasis*

	Patients without metastasis (***n*** = 86)	Patients with metastasis (***n*** = 10)	
	Mean (SD)	Mean (SD)	P**
Pain	28.9 (21.5)	35.1 (30.5)	0.66
Swallowing	28.6 (16.5)	38.8 (13.1)	0.06
Senses problems	13.8 (17.4)	25.9 (23.7)	0.10
Speech problems	24.4 (16.0)	44.4 (12.4)	0.001
Trouble with social eating	27.3 (17.4)	35.1 (24.5)	0.25
Less sexuality	19.9 (26.6)	35.1 (31.6)	0.09
Problems with teeth	42.6 (25.4)	61.7 (13.7)	0.02
Dry mouth/Sticky saliva	10.4 (19.7)	16.6 (20.4)	0.29
Body image	24.7 (21.2)	25.9 (21.5)	0.76
Shoulder pain	4.67 (14.4)	12.9 (23.2)	0.10
Skin problems	5.62 (12.5)	25.9 (35.1)	0.07
Anxiety	47.7 (30.2)	53.7 (30.9)	0.58
Trouble with social contact	9.7 (21.2)	37.0 (48.8)	0.003
Opening mouth	28.5 (31.2)	59.2 (27.7)	0.007
Coughing	8.8 (18.8)	33.3 (44.0)	0.04
Lymphedema	7.7 (16.8)	25.9 (27.7)	0.01
Weight loss	25.1 (31.4)	44.4 (28.8)	0.05
Problems wounds healing	11.5 (21.8)	25.9 (14.6)	0.005
Neurological problems	9.8 (20.7)	22.2 (23.5)	0.03

*Higher scores indicate greater symptoms

**Derived from Mann-Whitney U test

**Table 4:** The QLQ-H&N43 scores by stage of the disease*

	Stage I & II (***n*** = 34)	Stage III (***n*** = 26)	Stage IV (***n*** = 36)	
	Mean (SD)	Mean (SD)	Mean (SD)	P**
Pain	26.4 (23.5)	28.8 (15.3)	33.6 (26.1)	0.46
Swallowing	29.4 (15.7)	29.4 (19.4)	29.9 (14.9)	0.98
Senses problems	11.5 (16.1)	14.7 (20.4)	18.2 (17.6)	0.27
Speech problems	20.7 (17.1)	25.1 (15.5)	33.3 (15.3)	0.006
Trouble with social eating	23.0 (18.3)	26.4 (17.5)	33.8 (17.9)	0.09
Less sexuality	16.6 (26.0)	23.9 (28.6)	24.3 (27.5)	0.38
Problems with teeth	36.0 (23.9)	41.8 (25.7)	55.9 (22.1)	0.007
Dry mouth/Sticky saliva	9.3 (18.4)	10.9 (18.2)	13.4 (23.5)	0.68
Body image	19.0 (18.9)	26.7 (20.8)	29.4 (23.0)	0.11
Shoulder pain	3.8 (13.5)	5.5 (15.4)	6.7 (17.3)	0.74
Skin problems	4.7 (12.6)	6.3 (13.8)	11.4 (22.3)	033
Anxiety	43.4 (31.7)	47.4 (30.8)	54.3 (27.8)	0.39
Trouble with social contact	8.1 (14.5)	11.5 (24.8)	17.7 (31.6)	0.62
Opening mouth	21.5 (27.0)	37.1 (39.2)	37.5 (29.0)	0.08
Coughing	08.9 (17.7)	10.7 (21.2)	13.5 (29.1)	0.99
Lymphedema	03.8 (14.3)	11.1 (19.8)	12.5 (20.3)	0.10
Weight loss	20.2 (32.2)	24.3 (30.6)	36.5 (30.2)	0.05
Problems wounds healing	5.1 (12.2)	13.9 (20.6)	18.1 (26.4)	0.08
Neurological problems	6.4 (16.3)	8.0 (16.7)	18.2 (26.9)	0.08

*Higher scores indicate greater symptoms

**Derived from Kruscal-Wallis test

#### Convergent and divergent validity (discriminant validity)

The correlation between the EORTC QLQ-C30 and the EORTC QLQ-H&N43 scores was examined. As expected almost in all instances relevant subscales showed acceptable correlations and those not related exhibited low correlations. The detailed results are shown in Table 5.

**Table 5:** Correlation between the EORTC QLQ-C30 functioning and global quality of life and the EORTC QLQ-H&N43 scores*

	PF	RF	EF	CF	SF	GQOL
Pain	− 0.38 (0.004)	− 0.33 (0.007)	− 0.42 (< 0.001)	− 0.14 (0.18)	− 0.20 (0.68)	− 0.32 (0.002)
Swallowing	−.0.11 (0.29)	−.012 (0.27)	− 0.29 (0.005)	− 0.15 (0.15)	−.0.25 (0.02)	− 0.34 (0.01)
Senses problems	− 0.28 (0.006)	− 0.18 (0.07)	− 0.37 (< 0.001)	− 0.22 (0.03)	− 0.26 (0.01)	−0.17 (0.09)
Speech problems	−0.36 (< 0.001)	− 0.16 (0.12)	− 0.19 (0.06)	− 0.15 (0.16)	− 0.45 (< 0.001)	−0.43 (< 0.001)
Trouble with social eating	−0.20 (0.05)	− 0.25 (0.01)	− 0.37 (< 0.001)	−0.14 (0.19)	− 0.37 (< 0.001)	−0.23 (0.02)
Less sexuality	−0.53 (< 0.001)	− 0.35 (0.001)	− 0.24 (0.02)	−0.46 (< 0.001)	− 0.17 (0.11)	−0.40 (< 0.001)
Problems with teeth	−0.50 (< 0.001)	− 0.24 (0.02)	− 0.26 (0.01)	−0.04 (0.71)	− 0.27 (0.009)	−0.37 (< 0.001)
Dry mouth/Sticky saliva	−0.29 (0.006)	− 0.15 (0.16)	− 0.14 (0.18)	−0.17 (0.11)	− 0.09 (0.37)	−0.05 (0.62)
Body image	−0.07 (0.48)	−0.01 (0.92)	− 0.24 (0.03)	−0.18 (0.37)	− 0.22 (0.03)	−0.55 (< 0.001)
Shoulder pain	−0.30 (0.003)	− 0.51 (< 0.001)	− 0.15 (0.14)	−0.15 (0.14)	− 0.14 (0.16)	−0.41 (< 0.001)
Skin problems	−0.08 (0.43)	− 0.18 (0.08)	− 0.05 (0.60)	−0.04 (0.64)	− 0.18 (0.09)	−0.21 (0.05)
Anxiety	−0.27 (0.01)	−0.04 (0.69)	− 0.80 (< 0.001)	−0.04 (0.70)	− 0.05 (0.63)	−0.29 (0.006)
Trouble with social contact	−0.32 (0.002)	−0.16 (0.11)	− 0.10 (0.32)	−0.23 (0.03)	− 0.38 (< 0.001)	−0.31 (0.004)
Opening mouth	−0.32 (0.004)	−0.37 (< 0.001)	0.34 (< 0.001)	−0.24 (0.02)	− 0.21 (0.04)	−0.51 (< 0.001)
Coughing	−0.29 (0.005)	− 0.02 (0.88	− 0.01 (0.93)	−0.13 (0.21)	− 0.02 (0.8)	−0.13 (0.22)
Lymphedema	−0.30 (0.003)	−0.15 (0.14)	− 0.15 (0.14)	−0.15 (0.14)	− 0.15 (0.14)	−0.10 (0.33)
Weight loss	−0.25 (0.01)	−0.13 (0.22)	− 0.14 (0.16)	−0.13 (0.22)	− 0.24 (0.02)	−0.43 (< 0.001)
Problems wounds healing	−0.38 (< 0.001)	− 0.23 (0.03)	− 0.28 (0.007)	−0.25 (0.01)	− 0.41 (< 0.001)	−0.43 (< 0.001)
Neurological problems	−0.35 (0.001)	− 0.40 (< 0.001)	− 0.27 (0.01)	−0.33 (0.002)	− 0.35 (0.001)	−0.31 (0.003)

*Figures are Spearman’s rho coefficients and (*P* values). Negative signs are due to directions of scoring where higher scores on the EORTC QLQ-C30 represent better conditions and on the EORTC QLQ-H&N43 indicate greater symptoms. Abbreviations read as follows: *PF* physical functioning, *RF* role functioning, *CF* cognitive functioning, *SF* social functioning, *GQOL* global quality of life

### Internal consistency reliability

The Cronbach’s alpha coefficient for the multi-item scales of the QLQ-H&N43 ranged from 0.66 to 0.78 indicating that the internal consistency of the Azeri version of the questionnaire was acceptable. The results are shown in Table 6.

**Table 6:** Cronbach’s α for the EORTC QLQ-H&N43 multi-item subscales

	Number of items	Cronbach’s α
Pain	4	0.78
Swallowing	4	0.70
Speech problems	5	0.72
Social eating	4	0.66
Problems with teeth	3	0.73
Body image	3	0.69
Skin problems	3	0.72

## Discussion

The findings from this study indicated that the Azeri version of the EORTC QLQ-H&N43 is a valid instrument for measuring quality of life in head and neck cancer patients. The internal consistency in five out of seven multi-item subscales was good and only the Cronbach’s alpha for social eating (0.66) and body image (0.69) were slightly lower than acceptable threshold (0.7). Perhaps these could be due to cultural differences that exist among different nations. For instance, social eating is less common in Azerbaijan.

The authors of the original study that introduced the EORTC QLQ-H&N43 indicated that they analyzed the data in accordance with the EORTC Quality of Life Group QLG Module Development Guidelines. Thus, to retain an item in the module, they used 8 predefined criteria such as relevance, floor and ceiling effects, item difficulty, and compliance. They also used additional measures including the Cronbach’s alpha coefficient to assess internal consistency for multi-item subscales. The Cronbach’s alpha for hypothesized subscales they included in the module ranged from 0.77 to 0.87 that were well above acceptable values [17].

To the best of our knowledge contrary to expectation other than the original study, the current study is the second independent one that reports on psychometric properties of the QLQ-H&N43. The Serbian version of the QLQ-H&N43 was the first study that reported on psychometric evaluation of the questionnaire. The study included 170 patients and found good internal consistency for 5 out of 7 multi-item subscales. The study also showed that the questionnaire had acceptable validly (known groups validity) where patients who differed in type of laryngectomy, adjuvant therapy or 5-year survival scored differently in the expected directions [18].

We used known groups comparison for the validity purpose. As expected the Azeri version of the questionnaire well differentiated among patients who differed in stage and metastasis, although in most instances the differences among patients were not significant. For instance as reported in Table 5, only 8 out of 19 subscale showed statistically significant difference between the two groups, and in Table 6, only 2 out of 19 subscale showed statistically significant difference. One explanation for such observation might be related to the small sample size in each group. In addition since we analyzed the data for all types of oral cancers, therefore one might argue that if there was an opportunity to analyze the data for each sub-types of oral cancer, then it was possible to obtain significant results for all measures assessed. Finally as pointed out by Polit and Yang when performing known groups validity the direction of differences for scores among sub-groups (as hypothesized) are more important than statistical significant level values [19].

Interestingly the authors of the QLQ-H&N43 stated that the QLQ-H&N35 could still be used in ongoing or future studies if the investigators prefer to use this head and neck module version. However, they believe in studies investigating multimodal treatment or targeted therapies, the QLQ-H&N43 might be more suitable to detect differences between patient groups [17].

This study has some limitations. The sample size was relatively small. Secondly we did not perform test-retest analysis to investigate the stability. It seems that for using the Azeri version of the QLQ-H&N43 in future outcome studies we still need to perform further psychometric evaluations. However, one should note that the QLQ-H&N43 should be used with conjunction of the EORTC QLQ-C30 core cancer questionnaire, which is now available in Azeri version, too [20].

## Conclusion

The findings of this study suggest that in general the Azeri version of EORTC QLQ-H&N43 has satisfactory internal consistency reliability and validity, but additional psychometric evaluation is needed to draw firm conclusions.

## Data Availability

All data presented in this paper are available from the corresponding authors on a reasonable request.

## Abbreviations

EORTC QLQ-C30: The European Organization for Research and Treatment of Cancer core quality of life questionnaire
EORTC QLQ-H&N35: The European Organization for Research and Treatment of Cancer head and neck cancer specific quality of life questionnaire- 35 items
EORTC QLQ-H&N43: The European Organization for Research and Treatment of Cancer head and neck cancer specific quality of life questionnaire-43 items
